# Protocol for the assessment of the development of pragmatic competencies in early childhood (PDP-PI)

**DOI:** 10.3389/fpsyg.2024.1368321

**Published:** 2024-12-18

**Authors:** Cristina Junquera, Begoña Zubiauz

**Affiliations:** Department of Developmental and Educational Psychology, Salamanca University, Salamanca, Spain

**Keywords:** pragmatics, assessment, early childhood, communicative functions, language

## Abstract

**Introduction:**

The design of a Protocol for the Assessment of the development of pragmatic competences in early childhood (PDP-PI) and the preliminary data obtained in a comparative study in 3–5-year-old school children are presented.

**Methods:**

The design of the protocol is based on a model of global understanding of pragmatics that considers essential to include linguistic, intersubjective and social aspects in order to make an adequate assessment of development. Based on the taxonomies of communicative functions, four basic competencies are described (Interactional, Referential, Subjective and Figurative). These competencies make it possible to categorize most of the linguistic emissions in early childhood. The PDP-PI presents two novelties with respect to other assessment systems: (a) it allows detecting the degree of a skill development (not only the presence/absence), (b) it includes items to assess the comprehension of pragmatics. The PDP-PI was used with 40 students of kindergarten education, divided into three groups according to school year.

**Results:**

The results- Duncan’s *post-hoc* test-confirm the existence of significant differences between the different age groups in all competencies, except for referential competence. Construct validity was assessed by means of an inter-rater test (Fleiss’ Kappa = 0.76; Krippendorff’s Alpha = 0.76), and content validity was assessed by analyzing the correlations between the four competencies of the protocol. Significant correlations were found between all the competencies, except the relationship between the Referential and Interactional competencies.

**Discussion:**

The data obtained in this study are preliminary, but they show that there is an evolution in the management of pragmatic skills, throughout early childhood, toward more complex and context-appropriate interactions. It is necessary to advance in procedures that discriminate against this evolution to establish developmental profiles which favor the detection of pragmatic difficulties or disorders at early ages.

## 1 Introduction

Pragmatics is one of the most complex areas of language to define given its close relationship with inter-subjective skills and knowledge of the context of the interlocutors. The lack of consensus on a common approach to pragmatics hinders an integrated view of the existing scientific contributions, giving rise to molecular formulations and partial theories that explain specific processes and limit the creation of a global framework.

This research presents a global comprehension model of pragmatics in which the activation of three aspects, which feedback on each other, giving rise to a specific *speech act*, is considered essential:

– THE SELF AND COMMUNICATIVE INTENTIONALITY. The person who emits a speech act has a representation of himself that allows him to establish his beliefs, knowledge, intentions, and interests (Belinchón et al., 2000).
– THE OTHER: INTERSUBJECTIVITY AND THEORY OF MIND. The sender not only has a representation of his intentions, beliefs, and desires, but also a mentalistic representation of his interlocutor (second-order representation). This representation of the other includes information regarding aspects such as interests and beliefs, but also shared information, the relationship between the two and social distance (Baron-Cohen, 1995; Leslie, 1994), which would explain, for example, why the sender selects a topic of interest if he intends to please the interlocutor or tell only the end of a story if he knows that the beginning is known to both.
– THE CONTEXT: INFORMATION AND APPROPRIATENESS. Finally, to guarantee communicative success–although there can always be misunderstandings-, the sender must make a representation of the communicative context that includes the processing of information on referential, cultural and conventional aspects to be considered in that context.

Therefore, the elaboration of a message starts with the communicative intention of the sender who uses mentalistic information to adjust the linguistic selection; and this selection must conform to certain rules such as: the principle of cooperation (Grice, 1975), speech acts (Searle, 1969), the theory of misfortunes (Austin, 1962), or the rules of politeness (Brown and Levinson, 1987). The result is an initial message based solely on the sender’s perspective; but for the message to be successful it will need to be reworked considering the mentalistic information coming from the representations of the other and the context.

This conception of pragmatics has been supported by neuroimaging studies that place the areas of pragmatic processing in areas of the right hemisphere (Kupperberg et al., 2000) like those of mentalistic task processing (Kuhlen et al., 2015). There is sufficient data showing that during pragmatic performance both specific brain areas are activated (van Ackeren et al., 2012), as well as general processes (Apperly et al., 2005). In any case, neuroscientific research contributes to outline the close relationship between pragmatic and mentalistic skills.

However, despite the growing interest in the study of the acquisition of pragmatic skills in the early stages of language development, traditional language assessment tests are not very effective, since the multiplicity of skills that make up this domain make it incompatible with the use of standardized assessment methods (Conti-Ramsden et al., 1997). And in turn, the need for instruments that discriminate specific difficulties in this area is key for the early detection of disorders such as Social Communication Disorder (SCD) due to social reciprocity difficulties (Gibson et al., 2013; Loukusa et al., 2018) or pragmatic difficulties in Williams Syndrome (Díez-Ítza et al., 2016).

The question is how and with what instruments to assess the development of pragmatic skills at an early age to favor the detection of their difficulties and promote intervention from a normalized environment. From Psycholinguistics, we have proposals, although there is a premise that we should not forget: pragmatic symptomatology is not as objectifiable as other language components (González et al., 2015).

One of the most widely used approaches to assess pragmatic skills in early childhood is the Speech Act Theory (Searle, 1969) and its translation into classifications of Communicative Functions (Dore, 1974; Halliday, 1975; Bates, 1976; McShane, 1980; Ninio and Wheeler, 1984). These *classic* proposals continue to be representative lines of work in the exploration of the development of pragmatic skills since they are accessible and simple tools that do not require specific training of the evaluator and facilitate the elaboration of a general profile of communicative development. However, one of their limitations is that they evaluate skills in terms of the *presence or absence of* communicative functions, reducing the information obtained, because many functions can be satisfied with different levels of linguistic complexity. For example, a “demand” can be expressed with a simple gesture such as pointing or with an elaborate politeness formula. Therefore, it is not an all-or-nothing process, but a gradual process that denotes a differential use of possible communicative-linguistic formulations and the existence of levels of functional language development. This implies that the starting premise for assessing communicative functions is that they should not be studied as static elements, but that what is interesting is to check the degree of development, so that an adequate level for a three-year-old child may imply a delay in a five-year-old child.

Another limitation of these taxonomies is that they describe the *expressive* aspect of language without assessing *comprehension* or appropriateness to interaction or context. This conditions the assessment of development and prevents discrimination between performance or competence problems.

Taking as a starting point the model of global understanding of pragmatics (previously described) and the need to assess pragmatic skills between three and six years of age in order to detect possible difficulties, the aim of this work is to elaborate an assessment protocol, based on the taxonomies on communicative functions, that allows to collect data on the development of pragmatic skills in early childhood, including mentalistic skills and intersubjectivity. This evaluation system should make it possible to detect the existence of different levels of acquisition of the same pragmatic skill, showing that the management of the different communicative functions is a matter of degree and not of absence/presence. This age range was chosen for two reasons. Firstly, because most children in this age group attend early childhood centers and are likely to require a specific assessment for the detection of difficulties in language development. Secondly, because there are no instruments that systematically assess pragmatic development, in its expressive and comprehension aspects, so many difficulties may go unnoticed.

## 2 Materials and methods

### 2.1 Design

For the elaboration of the Protocol for the Evaluation of the development of pragmatic competences in early childhood (PDP-PI) the following objectives were followed: (a) to use as criteria to define the categories the objective of the act and the type of interaction in which the act is emitted; (b) to include the comprehensive aspect of Pragmatics; (c) to assume that the content of the instrument should include the main speech acts that develop between three-six years of age, without pretending to be an exhaustive taxonomy since it would not be operative.

### 2.2 Procedure for preparing the PDP-PI

#### 2.2.1 PHASE 1: Development of a pragmatic competence classification system

After carrying out a systematic analysis of the taxonomies on pragmatic competencies by Dore (1974); Halliday (1975), Bates (1976); Greenfield and Smith (1976), Togh (1987), Folger and Chapman (1978), Dale (1980); McShane (1980), Prutting and Kirchner (1983); Klecan-Aker and Lopez (1984), Ninio and Wheeler (1984), four categories were established: INTERACTIONAL, REFERENTIAL, SUBJECTIVE AND FIGURATIVE, integrated by different subcategories in their expressive and comprehensive aspects (See Table 1). The first three competencies are recurrent and are found in all the taxonomies reviewed, while Figurative is only referred to in Halliday (1975). This is explained by the fact that most of the classifications are aimed at very early ages in which intersubjectivity is usually valued as the expression of individuality and is therefore covered by Subjective competence.

**TABLE 1:** Taxonomy of pragmatic competencies.

Category 1. INTERACTIONAL COMPETENCE		
Speech acts aimed at directly influencing one’s own or others’ behavior and occurring in a shared action format.		
**Subcategories**		
**Management of outside assistance**	**Requests**	**Self-regulations**
Function: to influence the behavior of another		Function: to influence one’s own behavior
**Comprehension aspect: speech acts aimed at**		
- Respond verbally or behaviorally to the call for attention. - Attention check - Understanding a shift format	- Respond to requests (execution or protest) - Repair of shift in the face of lack of understanding	Absence of response to others’ self-instructions (understanding that it is not language directed at others).
**Expressive aspect: speech acts oriented toward**		
- Drawing the attention of others - Directing the attention of others - Check the attention of others	- Someone else to do something Types: - Direct requests: imperatives - Indirect requests: politeness formulas, questionsand suggestions.	- Planning and controlling one’s own behavior. Types: - Following verbal instructions - Self-instructions - Planning
**Category 2. REFERENTIAL COMPETENCE**		
Speech acts aimed at naming, describing or referring to a situation in the physical world to exchange information about it.		
**Subcategories**		
Denominate–describe	Relate concepts	Comment/share information
Function: labeling an element of reality	Function: to establish correspondences between different labels (inclusion, opposition, etc.)	Function: to exchange contents about reality (actions, activities).
**Comprehensive aspect**		
Decode messages that identify objects and situations	Extract information related to: - Opposition/equality - Membership	Decode messages referring to actions that occur in reality.
**Expressive aspect**		
Speech acts intended to label or describe reality.	Speech acts that include the management of relationships between concepts such as: - Antonymy/synonymy - Polysemy - Inclusion of categories	Speech acts referring to autobiographical information about knowledge and experience.
**Category 3. SUBJECTIVE COMPETENCE**		
Speech acts referring to the expression of the individuality of the self and the understanding of the subjectivity of others. They do not include objective aspects of reality; only intra- and interpersonal subjective elements: emotions, thoughts or interests.		
**Subcategories**		
Language linked to action	Language linked to the expression of individuality.	
Function: Statements that accompany actions and lack communicative intent.	Function: Express and understand intrapersonal or interpersonal subjective content.	
**Comprehensive aspect**		
Absence of response to playful expressions or similar responses from others	Speech acts or behaviors that involve the identification and understanding in other people of: - Physiological states - Desire or rejection - Emotions - Thoughts and beliefs	
**Expressive aspect**		
Use of humming, rhyming, and repetition during play activities	Personal expressions of the following states: - Physiological states - Desire or rejection - Emotions - Thoughts and beliefs	
**Category 4. FIGURATIVE COMPETENCE**		
Speech acts in which language does not represent reality directly. They are used in situations in which meanings are suspended for different purposes. Their use is linked to concrete context, and they have a high socio-cultural component.		
**Subcategories**		
Representational language	Lies and deceit	Non-literal language
Function: Emissions in which the suspension of meanings is linked to playful activities.	Function: Broadcasts in which meaning is suspended for the purpose of generating false beliefs in a competitor.	Function: Speech acts in which the literal meaning is suspended by causing a situation of divergence between the locution and the illocution of the speech act for aesthetic or humorous purposes, or with the intention of covering up or minimizing a thought or belief.
**Comprehensive aspect**		
Understanding the use of: - Roles - Fantasy - Representation	Understanding the interlocutor’s intentions in situations of: - Error - Instrumental lie - White lie	Understanding of the meaning, sense and intention of the - Metaphor - Hyperbole - Phrases
**Expressive aspect**		
Role-playing, fantasy and role-playing in play tasks	Lie management with intent to: - Make a profit - Avoiding damage	Use of non-literal statements such as: - Metaphors - Hyperboles - Phrases Other purposes (humorous, aesthetic, etc.)

The INTERACTIONAL category includes the earliest appearing linguistic functions, such as imperatives or intentionality. The language function underlying these speech acts is the regulation of behavior (one’s own or another’s) and is shown in joint action formats or as the structural basis of a communicative interaction.

The REFERENTIAL category is related to the narration of events and/or the organization of concepts and is linked to the interlocutors’ knowledge of the physical world, which allows speakers to share or demand information about reality. These skills require a high cognitive load since they do not necessarily refer to immediate contexts but make it possible to operate on elements that are not present.

SUBJECTIVE competence as an expression of individuality appears in some taxonomies as a personal use of language. It is composed of intra- and interpersonal identification and expression skills, such as personal language linked to action–frequent in play situations–and utterances where children express their individuality at a cognitive and emotional level (emotions, desires, interests, and thoughts). This type of content does not refer to knowledge of reality, but to the personal subjectivity of everyone. Therefore, they are not perceptible contents through the senses but must be inferred through mentalistic and representational skills.

FIGURATIVE competence includes the use of language to simulate, pretend or represent reality. These speech acts systematically violate the maxim of quality since they are not truthful utterances, and this lack of truthfulness is produced by a specific intention of the sender that can be playful (fiction game), interested (instrumental lie), or to express a complaint in an indirect way (irony). Understanding and using this type of statement requires advanced mentalistic skills that facilitate the understanding of the other’s intentions and the anticipation of their states or behavior.

Once the general categories were established, the skills for each one of them were determined, taking evolutionary development as a guideline; that is, skills that progress between three and six years of age. Finally, skills on the use and comprehension of non-literal statements (irony and metaphor) were incorporated, the assessment of which is increasingly proposed as necessary at these ages.

#### 2.2.2 PHASE 2: Elaboration of the evaluation tasks according to the interaction “Format”

Once the skills of each pragmatic competence were arranged, they were transformed into tasks, according to the notion of *format* of Bruner (1976), understood as a triadic interaction between the child, the adult, and an object. In this case, the selection of the object or action was made by adapting it to the type of responses that were intended to be induced.

For INTERACTIONAL COMPETENCE, an *action format was* chosen, since it requires the exchange of orders, requests and other speech acts, whose illocutionary force is oriented to the execution of actions. The task designed was the *joint construction of a puzzle*, taking as a reference the research of Bock and Hornsby (1981). This playful task allows the organization of turns of responses similar to those produced in a dialog.

For the REFERENTIAL COMPETENCE, a “*story reading*” *joint attention format was* chosen, since it allows to induce, in a natural way, informative nuances such as naming, description and comments. For example, the adult asks the child to describe one of the characters in the story (proposed situation), the child describes it (induced response) and the expression of perceptual description is assessed (assessed competence).

The selection of a task to promote the use and understanding of SUBJECTIVE COMPETENCE was a more complex process, since the expression of playful language and the manifestation of rejection required an *action format*, while the expression of needs and desires required an *attention format*. The task provided was *a game with dolls that* made the *mixed format* (action and attention) possible. As an example: a doll holding a backpack with many items is shown (proposed situation) to assess the child’s expression and understanding of interests and/or desires (assessed competence), for which the child is asked if these items are of interest or not, etc. (induced response).

For FIGURATIVE COMPETENCE, a *mixed format* was proposed through a task that includes a series of graphic vignettes that require the understanding and expression of everyday situations and the reasons that lead the characters to act in a certain way (humor, irony, lies, hyperbole) and that have been validated for the detection of mentalistic difficulties (White et al., 2009). The adaptations of Happé’s (1994) Strange Stories were: the inclusion of visual support, to favor the comprehension of the instructions in children of infant age (Aguilar et al., 2014), and the design of questions to assess the comprehension of the story, asking the child to explain what it means.

#### 2.2.3 PHASE 3: Development of the response coding system

The coding system of the responses to the tasks has different levels that allow for the assessment of the degree of acquisition of communicative skills, considering the milestones of pragmatic development and the competencies that underline the correct execution of each task.

**FIGURE 1:**
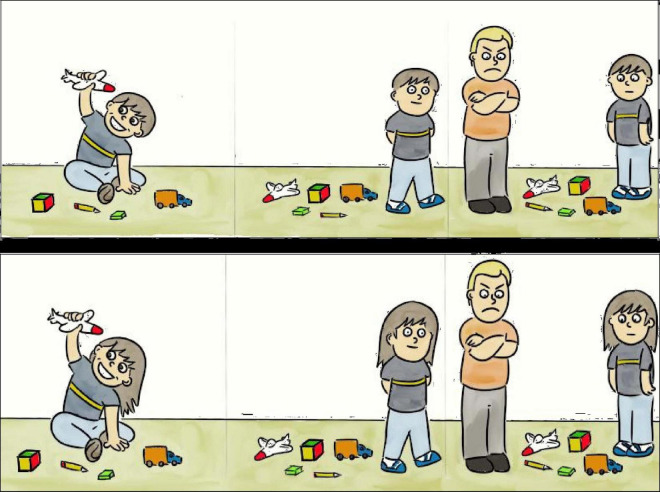
Scene to assess the understanding of irony.

For example, to assess the *comprehension of irony*, the evaluator first shows a picture with a scene (Figure 1) to the child with the following statement: “This boy has not picked up his toys. His dad comes into his room and says”, “How tidy your room is!” “What does what the daddy said mean?.”

The child’s response, as shown in Table 2, can have five levels: from no response or utterance of an unrelated response to understanding the sender’s intention in an ironic utterance. This breakdown of levels results in ordinary coding on a Likert-type scale. As this system entails difficulty in assigning the utterances issued in the different alternatives, examples of possible responses in each alternative, identified as “type responses,” were included in the protocol to facilitate correction.

**TABLE 2:** Levels of response and type responses of the task “Understanding Irony.”

Competencies involved	Sample responses
Failure to respond or issuance of an unrelated response	No response or not related
Literal understanding of the statement	That it is very well collected
Detection that there is a divergence between what is said and what is implied, interpreting it as an error.	He made a mistake; he said it backward.
Understanding that what is said is the opposite of what is implied without inferring the sender’s intention	That she’s dirty, she’s scolding you
Understanding of the sender’s intention in an ironic statement	What do you want me to pick up

#### 2.2.4 PHASE 4: PDP-PI structure

As can be seen in Table 3, the PDP-PI was composed of 30 items assessing the use and comprehension of pragmatic skills. Interactional Competence (six items) and Subjective Competence (10 items) are used in all age groups. The Referential Competence, composed of four items, is expected to be used in the two older age groups because the content included requires a cognitive and semantic level above three years of age to solve the tasks (Deák and Maratsos, 1998). The Figurative competence consists of 10 items and its use is foreseen in all age groups, except for two items of greater difficulty that require a meta-representational management typical of ages above 48 months (Recchia et al., 2010).

**TABLE 3:** Structure of the PDP-PI.

Competition	Number of items			Age (in months)
	**Expression**	**Comprehension**	**Total**	
Interactional	3	3	6	36–72
Referential	2	2	4	48–72
Subjective	5	5	10	36–72
Figurative	5	5	10	48–72 36–48 (only 8 items)

#### 2.2.5 Analysis of the adequacy of the content

To check the adequacy of the content, an expert judgment test was carried out,^1^ using a specific questionnaire to assess the appropriateness of each item in its category and the agreement between their ratings for each of the items. The results show a mean percentage of agreement of 82.64% (range 79.17: 89.58%). The Fleiss Kappa statistic was 0.76 and the Krippendorff Alpha was 0.76 indicating adequate reliability. Therefore, the judges identify the items with the competency they are assessing adequately, so that the content of the items appropriately assesses the competency for which they have been designed.

### 2.3 Participants

This study involved a sample of 40 students in the second cycle of infant education (see Table 4) attending a public school in the Community of Madrid. The exclusion criterion was to be detected as a special educational need student (developmental delays, difficulties in language development…). The participation of the students was voluntary, and the informed consent of the parents was obtained, as well as the approval of the school’s management team.

**TABLE 4:** Descriptive data of the sample.

Group	Course	Sample	Average age (in months)	Age range (in months)
1	1st EI	17 (10 boys; 7 girls)	43.47	38–49
2	2nd EI	15 (6 boys; 9 girls)	58.89	52–64
3	3rd EI	8 (4 boys; 4 girls)	69.25	64–76
Total		40	54.72	38–76

EI, early childhood education.

### 2.4 Method

For the inclusion of the participants in the sample, circulars were sent to the families informing them of the study and requesting the authorization of the parent/guardian for the participation of their children. Also, information sessions were arranged with the management and teaching teams to explain the objective of the research and the evaluation procedure. The students participated in the evaluation during the school day, so that the school calendar and the school’s own activities were respected.

All participants were evaluated with the PDP-PI individually, but to favor an ecological assessment and their collaboration, its application was integrated as part of the classroom routine. For this purpose, a corner, called “*play corner*” was set up at one end of the classroom, with a small table, two chairs and the PDP-PI materials (puzzles, puppets, pictures…). School materials were removed from the table so that there were as few distracting elements as possible. Students went to the play corner at the discretion of their tutors. The duration of each evaluation session was 30 minutes.

## 3 Results

### 3.1 Analysis of differences between groups

Table 5 shows the results obtained by Duncan’s *post-hoc* test (normality and homogeneity of variances were verified) which confirm that, in general, the scores of the three age groups are significantly different from each other: children in group 1 obtain the lowest scores, those in group 2 obtain average scores and those in group 3 obtain the highest scores.

**TABLE 5:** Duncan *post hoc* analysis of group difference (expressive and comprehensive aspects).

	Group	*N*	Media	SD	SEM	F
Interactional Competence						8.6970**
	1	17	1.25	0.255	0.062	
	2	18	1.05	0.25	0.059	
	3	8	1.47	0.198	0.07	
Referential Competence						2.0820
	2	18	0.83	0.409	0.096	
	3	8	1.06	0.21	0.074	
Subjective Competence						11.7560**
	1	17	0.8	0.395	0.096	
	2	18	1.13	0.465	0.11	
	3	8	1.64	0.207	0.073	
Figurative Competence						9.8030**
	1	17	0.45	0.317	0.077	
	2	18	0.8	0.426	0.1	
	3	8	1.13	0.35	0.124	

**Significance level 0.01.

**FIGURE 2:**
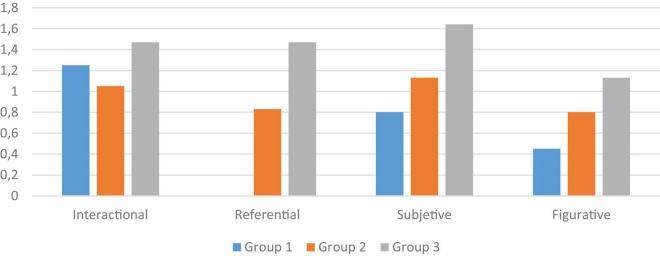
Differences between age groups in the assessed competencies.

In Interactional, Subjective and Figurative Competences, a significance index for mean differences of less than 0.01 is obtained (Figure 2). Therefore, in these tasks, older children show a better performance in the tasks, characterized by a better appropriateness of the utterances and a better understanding of the linguistic situations. In Subjective Competence these differences are especially marked, so that the development of these skills is especially discriminating in the three-six age range. The only competence in which no significant intergroup differences are obtained is Referential Competence (*F* = 2.0820). It should be remembered that this skill was only evaluated in children aged four and five years, and that it is the one with the fewest items.

### 3.2 Validity of protocol content

To confirm the content validity of the PDP-PI, the correlations between the competencies were analyzed. The results, shown in Table 6, show significant correlations between all competencies except Interactional and Referential. One possible explanation is that to share an action where referents are present in the communicative situation (and therefore shared by the interlocutors) it is not necessary to explicitly allude to these referents. In general, these results are a good indicator of content validity, since most of the competencies correlate with a bilateral significance level of 0.05, the relationship between Subjective and Figurative being much clearer.

**TABLE 6:** Correlation indexes between competencies.

	Interactional	Referential	Subjective
Interactional			
Referential	0.295		
Subjective	0.301*	0.483*	
Figurative	0.309*	0.486*	0.636**

*The correlation is significant at the 0.05 level (bilateral).

**The correlation is significant at the 0.01 level (bilateral).

### 3.3 Descriptive analysis of results

Given the novelty of the items and tasks that make up the protocol, we proceeded to a qualitative analysis, based on the percentage of responses obtained by each age group in the different items; this count would help to better compare and identify the differences in the performance of the three age groups evaluated. The scores are obtained by summing the responses of all the subjects in each group divided by the number of subjects. As for the mean score of each competency, it was extracted from the sum of the global results of all the tasks divided by the number of participants in the sample and the number of tasks that make up each competency. With these weighted averages, the differences between tasks and between groups can be analyzed qualitatively. The most significant results for each of the competencies are presented below.

First, the results obtained on the development of interactional skills (see Table 7) partially support the development of these skills associated with interactive contexts. The score achieved by the participants in group 2 prevents us from establishing a developmental profile, perhaps due to the high level obtained by the youngest age group (three-four years). Nevertheless, it is considered appropriate to continue with the evaluation of the development of more complex interactional skills, such as the expression and comprehension of requests, reformulation, and recovery of conversational turn, between three-six years of age, since significant differences in the management of these skills were observed in the different age groups.

**TABLE 7:** Mean scores obtained by the three groups in interactional competence.

Interactional skills		G1	G2	G3
Care management	C	1.52		
	E	0.88		
Mental-linguistic comprehension	Permission		0.64	1.62
	Attention		0.80	1.62
	Request		1.05	1.25
Requests	C	1.58	1.11	1.38
	E	1.08	1.61	1.81
Reformulation and shift repair	PR	0.88	1	1.12
	RF	1.52	1.41	1.5
Average score		1.25	1.05	1.47

G, group; C, comprehension; E, expression; RF, reformulation; RP, shift repair.

In addition, the qualitative analysis (see Table 8) performed on the comprehension task of *reformulating an utterance in a conversational context* shows that 70.59% of three-year-old participants can emit gestural responses (e.g., picking up a piece after being ignored) while 23.53% emit verbal responses. Therefore, assessing simpler formulas such as repetition (Manfra et al., 2016), would allow detecting early developmental levels of this skill involving verbal but not syntactic reformulation.

**TABLE 8:** Results of the item reformulation in Group 1 (three-four years).

Response levels	Counting	Percentage	Cumulative Percentage
1. No response or inadequate response	1	5.88	5.88
2. Nonverbal attempt to repair the shift	7	41.18	47.06
3. Gestual response “picking up piece”	5	29.41	76.06
4. Mitigated rephrasing “give it to me, please”	3	17.65	94.12
5. Protest demonstration, complaint, or reparation of the shift	1	5.88	100.00

Referential competence was the most complicated to define and operationalize. Perhaps because its mastery depends more on the subject’s knowledge of reality, and this knowledge derives more from semantic aspects (labels, vocabulary, etc.) and the development of cognitive processes (cognitive operations to establish relationships such as equality or difference between elements and categories), than from the more purely mentalistic aspects and understanding of the communicative context. Thus, when analyzing the errors committed in the tasks, it was detected that the problem was not due to the lack of skill in the process but rather to the content; in fact, the children explained that they could not answer because they “*did not know the words*.” Even so, there were significant differences in favor of the older students in all the skills assessed, which would support the progressive development of these skills (see Table 9).

**TABLE 9:** Mean scores obtained by groups 2 (four-five years old) and 3 (five-six years old) in the referential competence.

Referential skills			G 2	G 3
Reference communication	C			1.75
	E			0.93
Categorization	C	Categorical	0.80	1.63
		Unknown element		0.87
	E	Categorical	0.52	0.75
		Infer categories (1)	0.94	1.25
		Infer categories (2)		0.44
		Category Description		0.38
Analogies	C		1.05	1.5
Average score			0.74	1.05

G, group; C, comprehension; E, expression.

In Subjective Competence, it was found that children between three and six years of age can communicate, without difficulties, physiological states, primary emotions, and desires (preferences), data congruent with those found by Wellman and Bartsch (1994). Table 10 shows the differences between the three age groups, which are more striking than in the previous competencies, so that this competence seems to be particularly sensitive to development in these years. This may be due to two factors: either these are skills whose development is more abrupt at these ages, or the selection of tasks and levels of response, arranged in the protocol, has been very discriminative.

**TABLE 10:** Mean scores obtained by the three groups on subjective competence.

Subjective skills		G1	G2	G3
Physiological states	C	0.94		
	E	0.94		
Primary emotions	C	1.64	1.57	2
	E	1.64	1.54	2
Desires and interests	C	0.61	1.44	1.68
	E	0.73	1.30	1.75
Rejection	C	0.41	0.72	1.06
	E	0.31	1	2
Thoughts and beliefs	C	0.5	1.19	1.56
	E	0.26	1.16	1.82
Secondary emotions	C			1.43
	E			1.37
Average score		0.80	0.96	1.66

G, group; C, comprehension; E, expression.

The results on the expression and understanding of *primary emotions* corroborate that at three-four years of age (70%) children understand the emotional states of other people (Lenti et al., 1999) and that this acquisition would be defined at five-six years of age (producing a ceiling effect); therefore, from five years of age the evaluation should focus on the expression and understanding of *secondary emotions*. It is also evident that the expression and understanding of thoughts and beliefs is more limited at three-four years of age (11.76%), and its development begins around four-five years of age (44.44%) (Bartsch and Wellman, 1995).

In this study, for the *expression “rejection*,” it was necessary to generate a scenario that favored the use of alternative formulas to “*no*” in isolation, showing a progressive use of these linguistic formulas between the ages of three-four years (29.41%), four-five years (50%) and five-six years (100%).

As for figurative competence, according to the results obtained in this study, the development of figurative use of language is located between five-six years of age (see Table 11). However, it should be noted that some children between three and five years of age respond to these tasks, although their comprehension and use is partial.

**TABLE 11:** Mean scores obtained by the three groups in figurative competence.

Subjective skills		G1	G2	G3
Simulation	C	0.82	0.88	
	E	0.41	1.28	
Pseudo-lie	C	0.23	1.22	1.37
	E	0.14	0.66	0.56
Lie	C	0.11	0.69	1.5
	E	0.23	0.72	1
Metaphor	C	0.67	0.69	1.06
	E	0.79	1.16	1.5
Irony	C	0.64	1.08	1.72
	E	0.38	0.61	1.18
Phrases made	C		0.44	1
	E		0.22	0.75
Hyperbole	C			1.37
	E			1.12
White lie	C			0.62
	E			0.75
Average score		0.44	0.80	1.13

G, group; C, comprehension; E, expression.

^1^The participants were: a graduate in Hispanic Philology, an expert in teaching Spanish as a foreign language, a psychologist and educational counselor, and a speech therapist specializing in language disorders.

Regarding the understanding and use of pseudo-lies, majority of children aged three-four years (75%) do not understand these situations and do not say them either; while those aged five-six years understand them perfectly well (more than 76%) but do not express them (only 12% and not in a complex way). A similar profile appears among the four-five-year olds, although they show a greater mastery than the five-six year olds, an effect that may be due to the influence of the “social desirability” factor, in the sense that older children are more aware of those lies that should not be told, as opposed to the spontaneity of the four-five year olds.

Instrumental lying shows a progressive development (5% at three-four years; 27% at four-five years; and 75% at five-six years). In the older age group, it was found that many participants responded: “*it is wrong to lie*” or “*lying is wrong*.” In other words, although they understood the context and the objective of the task, they did not complete it because of the negative social burden associated with the use of lying. And with respect to the pious lie, the most striking result is that, although most of the five-six-year-old participants (75%) do not solve the task, 25% understand the intentionality of this type of lie and affirm that “they are capable of lying in order not to hurt someone.”

Considering the results obtained, it can be said that the development of metaphorical language begins at an early age (almost 50% of the participants aged three and four years understand a metaphorical expression) and whose progress will be continuous at later ages, beyond the age of six years. The age of inflection would be between four and five years of age, and by the age of six years most children (75%) show adequate mastery of this communicative use. The fact that children between three and six years of age seem to be more competent in making metaphors than in understanding them could be explained by the fact that in this period the verbal explanation of concepts resorts to analogy, so that these verbalizations adopt a form of simile, a pseudometaphor.

Regarding irony, the beginning of this figurative value of language appears around the age of four, but it becomes evident from the age of five-six: 100% of these children understand, and more than 75% are able to use language in an ironic sense, although with varying degrees of complexity.

Finally, within the figurative uses of language, it was found that the comprehension and expression of idioms undergoes a clear evolution between four and six years of age: 75% of children aged four -five years do not understand idioms and only 12% can make a sentence of this type. It is from the age of five-six that the development of this competence begins (more than 50% of children understand idioms and express at least one idiom).

## 4 Discussion

The present work is framed within the study of the development of pragmatic competence from a perspective in which the ability to tune in to the other person, read his or her mind and detect his or her interests are fundamental to explaining the adequacy of language to the communicative context.

### 4.1 Contributions

The main objective was to develop an instrument to assess the development of pragmatic competence in children between three and six years of age. For this purpose, an adaptation and adaptation of the taxonomies of communicative functions has been carried out in order to identify both the progressive acquisition of a competence and the mastery or difficulty in one or more specific skills.

The PDP-PI can contribute to complete the assessments in the pragmatic area by providing a different perspective and assessment methodology compared to other existing instruments such as the *Children’s Communication Checklist* (CCC-2) by Bishop (2003), the PEP-L (Romero et al., 2014), the *ABaCO* (Angeleri et al., 2012), or the *Pragmatic Observational Measure* (POM) (Cordier et al., 2014) that resort to information collection techniques such as direct observation or interviews with caregivers (parents and teachers). In this sense, the PDP-PI is integrated by performance tasks that favor the creation of natural contexts from the provision of *interaction formats* -games and/or narration of pictures or stories- as they avoid the negative effect of evaluation in favor of a comfortable context for children (Acosta et al., 1996; Botana and Peralbo, 2023).

Another of the main limitations of most pragmatic competence assessment instruments is that they only assess the expressive aspect. The lack of research on the comprehension of language uses represents a basic problem for traditional assessment in the elaboration of a complete developmental profile. However, this information is key in both the diagnosis and intervention of communicative-linguistic disorders. For this reason, the PDP-PI includes tasks to assess comprehension of utterances and contexts.

The last of the key elements of the PDP-PI is the response coding system that attempts to overcome dichotomous coding (presence/absence, appropriate/inappropriate) by favoring the determination of a more sensitive and exhaustive baseline.

The preliminary results obtained with the PDP-PI are congruent with the development of skills related to Communicative Functions (Nuñez and Riviere, 1994; Lee and Rescorla, 2002; Pascual et al., 2008; Airenti and Angeleri, 2011; Manfra et al., 2016). This implies that the type of tasks and the scoring method designed are relevant in the assessment of the pragmatic component of language. It is corroborated that the acquisition of Interactional Competence is circumscribed in this period and can serve as a reference for the detection of difficulties. The development of Referential Competence would be linked to the development of cognitive processes of interest for the detection of semantic-pragmatic difficulties. And the deployment of the Subjective function becomes evident from the age of 4 years, continuing from the age of 5 years, in parallel to the development of mentalistic skills, an age at which signs of figurative use of language are already detected.

A high correlation has been found between the four competencies assessed (Olivar et al., 2004), except for the Interactional and Referential Competencies. There are two possible explanations for these data: the marked pragmatic-semantic character underlying the nature of referential competence, which necessarily implies that the processing of linguistic and conceptual content can generate gaps; or that the reduction of skills that make up this competence in the PDP-PI, in favor of guaranteeing more pragmatic rather than mathematical functions, has affected the internal validity of this competence.

The correlation of Subjective competence with the rest of the competences is positive in all cases, highlighting the high correlation with Figurative competence. The data are consistent with research defending the high relationship between the use of mentalistic verbs and the resolution of theory of mind tasks (Lohmann and Tomasello, 2003) and the understanding of secondary emotions (Villanueva Badenes et al., 2000). The high correlation found between the Subjective and Figurative competencies refers to the delimitation of the pragmatic component of language and its close relationship with mentalistic skills.

### 4.2 Limitations and foresight

This research is not without limitations. The results obtained are not directly extrapolated due to the size and diversity of the sample. It would be necessary to use the PDP-PI with sufficiently large samples to address specific age profiles and to have robust data on the validity and reliability of the instrument.

A larger study with a more homogeneous population is also required to perform a factor analysis to determine the variance of each competency explained by each of the tasks, so that adjustments can be made to the design of the instrument.

Likewise, it would be interesting to check the existence of correlations with other pragmatic assessment instruments such as the CCC-2 in its adaptation to Spanish (Crespo-Eguílaz et al., 2016). This type of comparison is frequent in the validation of new instruments and provides data on their adequate design. For example, for the validation of the EDPRA (Botana and Peralbo, 2015), correlation with the Pragmatic Profile (Dewart and Summers, 1995) was used as an indicator of validity and reliability.

It would also be convenient to review the operationalization of the Referential Competence in the different age groups or if a more interactive format for assessing this competence can be considered. This would imply the reformulation of the tasks included in this competency and their comparison with other tests that include the assessment of semantics, to determine the coherence of referential tasks that involve an assessment focused on processes instead of contents.

In view of the results, a larger sample would allow preliminary scales of the development of subjective and figurative skills between the ages of 3 and 6 years; scales that would serve to check if there are differences in the developmental profiles of people with ASD (Autistic Spectrum Disorder), SLD (Developmental Language Disorder) or SCD (Social Communication Disorder).

## 5 Conclusion

The results obtained after the application of the PDP-PI are encouraging with respect to its validity and its use in educational or speech therapy centers, as well as its replication in the field of research on language pragmatics.

Although the reliability and validity data of the protocol are preliminary, they are sufficiently significant to consider the PDP-PI as a valid alternative in the assessment of the development of pragmatic competence in early childhood. The results obtained represent a change in the conception of the assessment of communicative competence. What is essential is not to identify which functions appear, but rather the degree of development that children demonstrate. This protocol provides a first initial evolutionary pattern of the development of Interactional, Referential, Subjective and Figurative competencies between three and 6 years of age, and not only in terms of language use but also in terms of comprehension of language functions.

Therefore, the Protocol for the Assessment of Pragmatic Competence in Early Childhood is not closed but is emerging as an active line of research in which data are still being collected and adjustments are being made.

Thus, the assessment of pragmatic skills in schools through the PDP-PI could be applied with three objectives: a preventive assessment, detection of students with suspected pragmatic difficulties, an optimizing assessment aimed at promoting or improving the pragmatic skills of students in general, and a diagnostic assessment in students with disorders or delays in the development of communication and language.

## Data Availability

The original contributions presented in this study are included in this article/supplementary material, further inquiries can be directed to the corresponding author.
